# Seasonal changes in mixed‐species bird flocks and antipredator information

**DOI:** 10.1002/ece3.6280

**Published:** 2020-04-24

**Authors:** Demeng Jiang, Kathryn E. Sieving, Estelle Meaux, Eben Goodale

**Affiliations:** ^1^ Guangxi Key Laboratory of Forest Ecology and Conservation College of Forestry Guangxi University Nanning China; ^2^ Department of Wildlife Ecology & Conservation University of Florida Gainesville FL USA

**Keywords:** antipredation behavior, mixed‐species groups, mobbing, nuclear species, tropical seasonality

## Abstract

Animals acquire information produced by other species to reduce uncertainty and avoid predators. Mixed‐species flocks (MSFs) of birds are ubiquitous in forest ecosystems and structured, in part, around interspecific information transfer, with “nuclear” species providing information that other species eavesdrop on. We hypothesized that in a seasonal tropical forest, the amount of information produced by birds about predation would be dynamic and particularly would decrease inside MSFs when the nuclear species leave MSFs to breed. We obtained baseline information on MSF encounter rate and species composition along established sampling routes over 9 months near the Sino‐Vietnamese border. We also conducted three experiments to quantify information produced by different species in response to typical predator encounters, including a moving predator stimulus presented inside of MSFs, and a stationary predator model presented both inside and outside of MSFs. MSFs were much less frequent in the breeding season with fewer individuals of the nuclear species, David's Fulvetta (*Alcippe davidi*), participating, though the diversity of other species remained stable. Fulvettas were the dominant producer of alarm‐related information both to the moving and stationary stimuli in MSFs and were also among the most active mobbers to stimuli presented outside of MSFs. In the breeding season, they tended to call less to the moving stimulus, and substantially fewer individuals responded to the in‐flock stationary stimulus. Other species increased their own information production at stationary predator stimuli (inside and outside of MSFs) during the breeding season, perhaps due to their increased investment in offspring during this time. Yet even during the breeding season, David's Fulvetta remained the highest producer of information about predators in MSFs. Hence, while we show that information production in MSFs can be somewhat dynamic, we describe a continually asymmetric communication system, in which a nuclear species is important to the whole community.

## Graphical abstract

On the northern border of the tropics, we studied the seasonality of mixed‐species bird flocks and vocal information production, in response to a moving object in flocks (hawk‐flock), an owl model in flocks (mob‐flock), and the same model outside of flocks (mob). We found information from the nuclear species, David's Fulvetta (DAFU), tended to decrease in the breeding season in hawk‐flock experiments, and there were fewer responding individuals in mob‐flock experiments. In contrast, there were more species (other than DAFU) that responded during the breeding season in the mob‐flock experiment, and these species produced more information in the mob experiment.

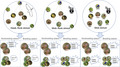

## 1 INTRODUCTION

Animals acquire information produced by other individuals to reduce uncertainty and avoid predators (Dall, Giraldeau, Olsson, McNamara, & Stephens, 2005; Danchin, Giraldeau, Valone, & Wagner, 2004; Schmidt, Dall, & van Gils, 2010). Although social information is often obtained from conspecifics, information from heterospecifics can also increase animals’ fitness, and under some circumstances, it may be as or more valuable than that obtained from conspecifics (Seppänen, Forsman, Mönkkönen, & Thomson, 2007; Sridhar & Guttal, 2018). Interspecific information transfer about resources or predators has been documented in many taxa (Farine, Aplin, Sheldon, & Hoppitt, 2015; Goodale, Beauchamp, Magrath, Nieh, & Ruxton, 2010; Ibanez‐Alamo et al., 2015; Sridhar & Guttal, 2018). In particular, animals have been shown to be able to use detailed information about the risk and characteristics of predators encoded in the signals of heterospecifics (Huang, Sieving, & St Mary, 2012; Rainey, Zuberbühler, & Slater, 2004; Templeton & Greene, 2007). Given its uniquity, interspecific information transfer is thought to have widespread consequences for the social organization and dynamics of animal communities (Gil, Hein, Spiegel, Baskett, & Sih, 2018; Goodale et al., 2010).

Mixed‐species flocks of birds (MSFs), a subset of avian assemblages worldwide and particularly prevalent in terrestrial forested habitats (Goodale, Beauchamp, & Ruxton, 2017; Zou et al., 2018), are an example of a community shaped by interspecific information transfer (Goodale et al., 2010). For most MSF systems, there appear to be certain “nuclear” species that are more important for MSF formation or maintenance than others (Moynihan, 1962; Zou et al., 2018). These species are usually active and noisy (Hutto, 1994), gregarious (Goodale & Beauchamp, 2010) and are more likely to be cooperative breeders than other species (Sridhar, Beauchamp, & Shanker, 2009). Some costly signals such as alarm calls, which might attract the attention of predators (Klump, Kretzschmar, & Curio, 1986; Klump & Shalter, 1984; Krams, 2001), may be made by these species because they can indirectly benefit by informing their kin or mates of risks (Maynard Smith, 1965). While many signals may often be directed toward conspecifics, nuclear species may also sometimes direct their signals toward heterospecifics, such as when alarm calls could provoke a scattering response that would be confusing to a predator (Charnov & Krebs, 1975), when recruiting other individuals to a food resource to dilute predation risk without greatly increasing competition (Farine et al., 2015; Hillemann, Cole, Keen, Sheldon, & Farine, 2019; Seppänen et al., 2007), or manipulating other species (Flower, Gribble, & Ridley, 2014; Goodale & Kotagama, 2006). Regardless, other species may join or follow nuclear species because of the information that they produce (Goodale et al., 2020). Indeed, following species may accrue more benefits in MSFs then do nuclear species (Gentry et al., 2019; Hino, 1998). Nuclear species can also affect the fitness of followers: when nuclear species are removed experimentally from MSFs, the body condition of following species can suffer (Dolby & Grubb, 1998) and followers may become more risk adverse (Martínez, Parra, Muellerklein, & Vredenburg, 2018). Hence, most MSF systems show an asymmetric pattern of information production and use.

Another kind of grouping phenomenon that is often centered around interspecific information transfer is avian mobbing. In mobbing, birds surround a stationary predator, reducing its ability to make surprise attacks and harassing it so that it often leaves (Curio, 1978; Pavey & Smyth, 1998; Pettifor, 1990). Compared with alarm calls, which are difficult to localize, mobbing calls are highly detectable (Marler, 1955). Indeed, many bird species have similar mobbing calls and are attracted toward each other (Dutour, Lena, & Lengagne, 2017; Jurisevic & Sanderson, 1994; Langham, Contreras, & Sieving, 2006; Nocera, Taylor, & Ratcliffe, 2008). Perhaps this is because recruiting a heterospecific to mob might be as effective in gaining a partner to dilute risk or harass a predator as recruiting a conspecific, and be less costly to the signaler (i.e., not risking a kin or a mate). Interestingly, many nuclear species of MSFs are also important mobbing initiators, perhaps because they are preadapted to be information providers by being gregarious, social species (Goodale et al., 2020). For example, the Black‐capped Chickadee is a MSF nuclear species that is also important as a mobbing initiator (Hurd, 1996; Nolen & Lucas, 2009). Hence, the importance of nuclear species may extend well beyond MSF systems (also see Mönkkönen & Forsman, 2002), especially among species (birds and mammals) that readily utilize heterospecific information produced vocally by nuclear species (Jones & Sieving, 2019; Schmidt, Lee, Ostfeld, & Sieving, 2008).

Both MSF systems and mobbing assemblages can be strongly seasonal. In temperate systems, MSFs usually form in late summer and during migration, and then continue through the winter until they breakup as breeding season starts (Morse, 1970; Rodewald & Abrams, 2002). Although some tropical MSF systems are very stable (e.g., Munn & Terborgh, 1979), others can change across breeding and nonbreeding seasons in their composition and size (e.g., Develey & Peres, 2000; and Tubelis, Cowling, & Donnelley, 2006). Unfortunately we know of no research on the effects of nuclear species breeding on MSF systems, except that of Jayarathna, Kotagama, and Goodale (2013), who found an MSF system in Sri Lanka to be stable even during the breeding season of the nuclear species. Multi‐species mobbing in temperate bird communities (evoked naturally or via experimental stimuli) usually peaks in nonbreeding winter seasons (Dagan & Izhaki, 2019; Dutour, Cordonnier, Léna, & Lengagne, 2019). Temporary mobs may also form during breeding seasons, but typically these revolve around distressed parents fending off nest predators, and fewer species participate (Shedd, 1982; Shields, 1984; Smith & Graves, 1978; Zimmermann & Curio, 1988).

A key question for bird communities in general, and especially for seasonal tropical systems, is whether the production of information of nuclear species or mobbing initiators changes over the annual cycle. To our knowledge, there have been no studies on the seasonality of heterospecific information production by nuclear species or following species. It is possible that if the information available from nuclear species was to decline, other species might make sure their conspecifics remain informed by producing information themselves (an argument made in a slightly different context by Goodale & Kotagama, 2005). Alternatively, other species’ information production may not be connected to that of the nuclear species’, but may be influenced by their own breeding ecology, and particularly the presence of young (Ibanez‐Alamo et al., 2015).

We examined seasonal variation of heterospecific information regarding predators (mobbing and attack contexts) in a forest on the northern boundary of the tropics that is of intermediate seasonality between tropical and temperate systems. First, we documented seasonal changes in the characteristics of the MSF system along transects (e.g., MSF encounter rate, MSF size), and then conducted three types of experiments mimicking common predator encounters to elicit key antipredator information. To simulate a surprise attack by a flying hawk (generating alarm calls) inside of MSF, we used a moving object (Goodale & Kotagama, 2005) and we stimulated mobbing responses with a small owl model (Hua & Sieving, 2016), presented both inside and outside of MSFs. We hypothesized that while the nuclear species would serve as the central information producer of MSFs, the marked seasonality of our system could cause fluctuations in both flocking and in the availability of social information. The gregarious nuclear species of this system, David's Fulvetta (*Alcippe davidi*), is not thought to be a cooperative breeder (Jiang, Zhou, Jiang, & Chen, 2013; Zhou, 1989), and so we expected that breaking into breeding pairs would disrupt their flocking system. We predicted that: (a) MSF encounter rate would decrease in the breeding season, with the participation of nuclear species decreasing as it engages in breeding activities, (b) behavioral responses to predator stimuli would be dominated by nuclear species both inside and outside of MSF, (c) nuclear species would decrease the information provision during the breeding season in MSF as they participate less in MSF, and (d) other species would increase their information production in the breeding season, either in compensation from the loss of information from the nuclear species, or because of their own investment in offspring.

## 2 METHODS

### 2.1 Study site and transect selection

The study was conducted at Nonggang National Nature Reserve, which is located near the Sino‐Vietnamese border, in the southwest of Guangxi Zhuang Autonomous Region, southern China (22°47ʹN, 106°95ʹE). Nonggang is on the northern edge of the tropics and contains well‐protected limestone karst monsoonal rain forests. The wet season begins in April and continues to September. Breeding season for birds starts in late March and ends in middle July, peaking in early May (Jiang et al., 2013).

Six 1‐km transects were placed on infrequently traveled, unpaved roads (between 1 and 2 km apart). We visited all transects once in January 2017 and then visited transects twice each month from February to July 2017, once in the morning and once in the afternoon of a different day, with the order of visits to the various transects systematically varied every month. We continued observation in November 2017 and February 2018, but for these 2 months, we did one visit for bird censusing, but two visits for behavioral experiments. For each transect visit, we did bird censusing in one direction and then conducted the behavioral experiments as we came back in the other direction (with the exception being the transects in November 2017 and January 2018 on which we only did experiments in one direction). We divide the breeding cycle into two categories: breeding season (March to July) and nonbreeding season (November, January and February).

### 2.2 Measurements of MSF encounter rate and species composition

During a transect walk, two observers moved slowly (0.75–1 km per hour), recording all birds detected within 50 m of the transect, starting at 8:30 a.m. or 3:00 p.m. All birds were noted as participating or not participating in an MSF, which was defined as two or more species moving in the same direction for more than 5 min (Goodale et al., 2009). After encountering an MSF, we spent a maximum of 15 min to record its composition, defining a complete MSF as one in which no new species were seen over the last 5 min of the observation.

### 2.3 Behavioral experiments

In this study, we conducted three types of experiments that reproduce different contexts that are commonly encountered by forest birds. First, we simulated a raptor attack by throwing a stick over MSFs (“Hawk‐flock” experiment), which generates an immediate but fleeting response. This experiment was modeled after that of Goodale and Kotagama (2005), who showed that birds will alarm call to any large, fast‐moving object, even a stick, at least initially (though such alarm calls are usually shorter than those made to actual predators). Second, we exposed MSFs to a model of a perched owl and its call (“Mob‐Flock” experiment). We expected MSF members to quickly discover the model and develop a protracted mobbing response. Third, we used the same owl model when there was no MSF within 50 m (“Mob” experiment). This experiment assessed species that were solitary or in monospecific pairs or groups and asked whether they would be willing to find and mob an owl.

To avoid repeat sampling of the same individuals, we aimed to conduct only one trial of each kind of experiment (Hawk‐flock, Mob‐flock, Mob) per transect visit and different experiments on the same transect were conducted at locations at least 250 m apart. Some individuals were undoubtedly resampled at different times of day within a month and in different months. However, we think the overall rates of resampling individuals were not that high, because MSFs were large and there were multiple MSFs present at each of the transects (see Results). Moreover, there was no evidence of habituation: for example, the number of species/individuals that responded was as high at the end of the experimental period as it was in the beginning. Although we aimed for one trial of each type of experiment per transect visit, low MSF encounter rates precluded conducting many experiments inside MSFs in the breeding season.

#### 2.3.1 Hawk‐flock experimental protocol

The experiment started by encountering and observing an MSF for 10–15 min, and recording the composition. The observers aimed by the end of this period to be approximately 15 m from the edge of the MSF and ensured that the birds’ vocalizations indicated that the MSF was not disturbed by them. At this point, one observer then audio‐recorded 30 s of the vocalizations of the MSF, using a Marantz PMD 671 digital recorder and a Sennheiser ME62 omnidirectional microphone embedded in a Telinga parabola, and making the recordings with a sample rate of 44,100 Hz (24 bit). After 30 s of baseline activity had been recorded, the other observer threw a stick (approximately 0.5 m long, 5 cm diameter) over the center of the MSF, aiming for a height on the throw of approximately 6 m. Stick throws were exclusively completed by DJ, who practiced so as to be consistent in the speed and distance of the throw. The recording was continued only for 90 s, because of the fleeting response to this kind of stimulus.

#### 2.3.2 Mob‐flock and mob experimental protocol

For these two types of experiments, we presented a model, associated with playback from a speaker. We used a decoy Collared Owlet (*Glaucidium brodiei*), a species that is common at the study site and can be active in the daytime. We made two models of this out of styrofoam, covered in painted chicken feathers, and with yellow glass eyes. We made four playback tapes from recordings found on the website Xeno‐Canto (https://www.xeno‐canto.org/; selecting A grade recordings made as close as possible to the study site). Playback tapes were made from 30 s segments of these recordings with high vocalization rate, followed by 30 s of silence, repeated five times. For any trial, we decided randomly which of the two owl models and which of the four playback tapes were used.

The mob‐flock and mob experiments used very similar protocols. The mob‐flock experiment started with 10–15 min observation of the MSF, to habituate the birds to the observers’ presence and to judge MSF composition. The predator model was then setup on a pole 3 m tall, with a speaker (version WA‐35, JTS Professional Ltd) located beneath that on the ground, and the pole was situated around 10 m from the edge of the MSF (so our activity would not attract the attention of the birds). We recorded 30 s of baseline vocalizations, similar to the hawk‐flock experiment, and continued until 90 s after playback ended. A species was scored as to whether it vocally responded—making alarm calls (later verified with the recordings; see below) within 30 m of the speaker—and/or whether it made an approach toward the playback speaker while displaying antipredator behavior (changing perch position frequently, or conspicuous head‐turning and searching). We also estimated the number of individual David's Fulvettas that vocally responded, given that earlier work highlighted the importance of this species to MSF vigilance (Chen & Hsieh, 2002; we did not do this in the hawk‐flock experiment because the behavioral response in that experiment occurred nearly instanteously, making such estimation difficult). The mob experiment consisted of the very same procedures as the mob‐flock experiment, but ensuring that MSFs were absent within 50 m.

#### 2.3.3 Control experiments

We also conducted control experiments for the playback experiments. As models, we used two specimens of Oriental Turtle Dove (*Streptopelia orientalis*), a nonpredatory species that is of relatively similar size to the Collared Owlet and also has low‐pitched calls. Four tapes for playback were made based on recordings downloaded from the website Xeno‐Canto. Experiments were performed when MSFs were present and also when MSFs were absent, following the same protocols as the mob‐flock experiment and the mob experiment, respectively.

### 2.4 Scoring of recordings

We created spectrograms using Raven 1.3 (Cornell Laboratory of Ornithology) with a Hamm Window and FFT between 512 and 1,024. We scored the recordings for the presence of alarm calls, with the definition of an alarm call being a change from silence during the baseline period to vocalizing, or a change in call type from the baseline period (again following Goodale & Kotagama, 2005). For all species, the call types used in the period after playback were dominated by certain vocalizations that we also noted in vocal responses to real predators (two observations of raptors) and initial reactions of the birds to the human observers. Latency of the alarm call was measured as the time between the start of the experiment (presentation of the stimulus) and the start of the alarm call, and duration was measured as the first to last notes of alarm within the recording.

### 2.5 Statistical analysis

We constructed generalized linear mixed models (GLMM), using the “lme4” package (Bates, Maechler, Bolker, & Walker, 2015) in R (version 3.6.0, R Core Team 2019). A table of the different models and variables in presented in Table 1. Briefly, most analyses investigated the influence of fixed factors of seasonality and/or species identity on the response variables, and for all models transect was incorporated as a random variable to account for the repeated visits to the same transect in different months. Time of day was found not to be important in preliminary analyses, and thus not included. Most models used a normal distribution and thus were technically linear mixed models (LMM), but we used GLMMs with binomial distributions when analyzing whether species responded vocally for the hawk‐flock experiment or whether species approached in the mob‐flock experiment. Square root transformations of the response variables were used to minimize departures from normality, as visually assessed by residual plots. Multi‐comparisons were conducted with the “multcomp” package for pair‐wise comparison (Hothorn, Bretz, & Westfall, 2008).

**Table 1:** Models included in the analysis of the seasonal variation in MSFs and the results of the three types of experiments. Transect was incorporated as a random factor for all models. In addition to these models, for the three experimental types, we tested whether each species that responded (using only those species that responded in over 10 trials) differed seasonally in its response probability, latency and duration (see Table 3). Results for the variable of species identity include the number of contrasts between species pairs that were significant. The coefficient is that which had the least significance among all significant contrasts; David's Fulvetta (DAFU) is the reference. *p*‐values for this variable are from Tukey's HSD‐corrected multiple comparisons. A positive value for species identity indicates that the species had a greater value for that variable than DAFU. A positive value for seasonality indicates nonbreeding was higher than breeding season. LMM = linear mixed model; GLMM = generalized linear mixed model

	Response variable	Method	Predictive variables									
			Season^a^				Species					
			Coefficient	*SE*	χ^2^	*p*	*df*	Significant contrasts	Coefficient	*SE*	χ^2^	*p*
Flock encounter rate and composition	Flock encounter rate	LMM	1.19	0.26	19.7	<.001	–	–	–	–	–	–
	Number of flocking species	LMM	0.39	0.35	1.1	.28	–	–	–	–	–	–
	Number of individuals of DAFU	LMM	2.07	0.54	13.9	<.001	–	–	–	–	–	–
	Number of individuals other than DAFU	LMM	1.80	0.93	3.8	.052	–	–	–	–	–	–
Hawk‐flock experiments	Richness of responding species	LMM	0.059	0.20	0.1	.76	–	–	–	–	–	–
	Vocal response probability	GLMM	0.52	0.43	1.6	.21	10	10	−1.97	0.60	83.6	.029
	Vocal latency	LMM	−0.47	0.31	2.4	.12	1	1	2.72	0.32	43.6	<.001
	Vocal duration	LMM	0.02	0.51	0.01	.98	1	1	−2.14	0.52	14.7	<.001
	Number of vocally responding DAFU individuals^b^	–	–	–	–	–	–	–	–	–	–	–
Mob‐flock experiments	Richness of responding species	LMM	−0.23	0.13	3.0	.082	–	–	–	–	–	–
	Approach response probability	GLMM	−0.66	0.39	2.8	.092	6	6	−2.95	0.95	64.6	.030
	Vocal latency	LMM	0.02	0.66	0.1	.97	3	3	2.52	0.83	49.3	.013
	Vocal duration	LMM	0.71	0.92	0.63	.4	3	2	−2.86	1.18	10.0	.017
	Number of vocally responding DAFU individuals	LMM	0.69	0.21	10.2	.002	–	–	–	–	–	–
Mob experiments	Richness of responding species	LMM	−0.13	0.11	1.3	.27	–	–	–	–	–	–
	Approach response probability^c^	–	–	–	–	–	–	–	–	–	–	–
	Vocal latency	LMM	−2.97	1.0	8.9	.003	5	3	3.85	1.47	15.5	.01
	Vocal duration	LMM	−0.08	0.66	0.1	.9	5	4	−2.04	0.97	17.0	.038
	Number of responding DAFU individuals	LMM	−0.21	0.21	1.1	.3	–	–	–	–	–	–

^a^ Degrees of freedom for the variable “season” were all 1.

^b^ We did not take estimates of the numbers of individual fulvettas vocally responding in the hawk‐flock experiment because the rapidity with which the behavioral response occurred made such estimates difficult.

^c^ Approach response probability was not applicable for the mob experiment because there were no birds in MSFs present at the start of the experiment to respond. However, we did compare species as to whether the proportion of trials in which they responded by approaching changed seasonally (see Table 3).

#### 2.5.1 Analysis of MSF encounter rate and composition

All MSFs were included in the analysis of MSF encounter rate, but only complete MSFs were used for the analysis of MSF composition. Additionally, to reduce the influence of rare species, we excluded species that participated in <5% of MSFs. We ran separate models for the different characteristics of MSFs: flock encounter rate, number of flocking species, number of individuals of David's Fulvettas, and number of individuals of other species. In these models, seasonality was the only fixed predictor variable (two levels, nonbreeding and breeding season).

#### 2.5.2 Analysis of experimental results

We analyzed responses for the three different kinds of experiments separately, but most analyses were applicable to all of the experimental types (see Table 1 for a table of the analyses). A first analysis was at the community level. The response variable was the number of species that responded per trial, and the fixed predictor variable was seasonality (single‐factor model). A second analysis investigated the effect of seasonality and species identity (two‐factor model) on the characteristics of species' responses, and was repeated for the different response variables (vocal response probability for hawk‐flock experiment, approach probability for the mob‐flock and mob experiments, and the latency and duration of vocal responses for all experiments). To increase the statistical power to observe differences between species or seasons, we included a species in the dataset only if it met the threshold of being present (in analysis of response probability) or vocally responding (in analyses of the characteristics of vocal responses) to at least 10 trials in the two different seasons combined. We excluded the interaction between seasonality and species identity from the full model because it was never significant and caused convergence issues for some models. Two‐factor models were simplified by removing one variable, if it was not significant.

A third analysis investigated each species’ responses separately and determined whether there was seasonal change (single‐factor model) in the response probability, latency or duration of each species’ responses. For these analyses, we tested species as long as they were present (in analysis of response probability) or vocally responded (in analysis of the characteristics of the vocal responses) to at least five trials in each season. A fourth analysis explored whether the number of individual David's Fulvettas that responded changed seasonally (single‐factor model; excluding the hawk‐flock experiment, for which these data could not be estimated).

Although most of the models applied to all experimental types, approach probability was not conducted for the mob experiment because we did not have information on what species were present before the stimulus (whereas hawk‐flock and mob‐flock experiments included observations of MSF composition). To understand seasonal change in the mob experiment, we determine whether the proportion of trials in which a species approached changed between the two seasons with Fisher's exact tests. We also used a Fisher's exact test to understand the seasonality of the approach of a nonflocking species during the mob‐flock experiment (as it was not present before trials and thus its probability to respond could not be calculated).

The power of our analyses was often low, based both on sample size and the fact that most of our analyses were on the species level. Seasonal change may be obscured for gregarious species because even if the number of responding individuals was lower in one season, as long as one individual responded, the whole species was rated as responding. For these reasons, we consider statistical results with *p*‐values <.05 as significant, but also discuss “tendencies” with *p*‐values ≥.05, but <.10. Mean values are shown ± *SD*.

## 3 RESULTS

### 3.1 MSF encounter rate and composition

In total, we made 83 transect visits, including 56 in the breeding season. Visits were relatively evenly spread among the six transects, which were visited an average of 13.8 ± 1.3 times. We encountered a total of 93 MSFs, 70 of which were considered complete; of these, 35 were in the breeding season. Although the overall mean of MSFs per transect visit was low (average = 1.1 ± 1.2), there were multiple MSFs present on each transect on at least some visits (the maximum number of flocks seen on a transect was 2, 3, 3, 3, 4 and 5 for the six transects). There were 18 species that participated in more than 5% of MSFs (Table 2), and on average 4.6 ± 1.4 species and 10.8 ± 5.0 individuals per MSF. David's Fulvetta was the species in the highest percentage of MSFs in both seasons.

**Table 2:** The frequency by which species participated in MSFs, and the seasonal variation in this measure. All species seen in more than 5% of the 70 MSFs are listed

Species	Common name	Scientific name	Total number of MSF in which present	Number of individuals in MSF (mean ± *SD*)	Flocking frequency in breeding (% of 35 MSF)	Flocking frequency in nonbreeding (% of 35 MSF)
DAFU	David's Fulvetta	*Alcippe davidi*	60	3.4 ± 2.3	0.83	0.89
PSTB	Pin‐striped Tit‐babbler	*Macronus gularis*	53	3.3 ± 2.1	0.60	0.74
RCBA	Rufous‐capped Babbler	*Stachyridopsis ruficeps*	35	2.4 ± 1.3	0.57	0.43
WBER	White‐bellied Erpornis	*Erpornis zantholeuca*	25	2.3 ± 1.0	0.29	0.43
BIWA	Bianchi's Warbler	*Phylloscopus valentini*	18	1.5 ± 1.2	0.14	0.37
LLWA	Limestone Leaf‐warbler	*Phylloscopus calciatilis*	18	2.3 ± 1.3	0.37	0.14
SBSB	Streak‐breasted Scimitar Babbler	*Pomatorhinus ruficollis*	16	1.5 ± 0.7	0.23	0.23
WTFA	White‐throated Fantail	*Rhipidura albicollis*	15	1.0 ± 0.0	0.11	0.31
CCWA	Chestnut‐crowned Warbler	*Phylloscopus castaniceps*	13	1.6 ± 1.0	0.14	0.23
YBEWA	Yellow‐bellied Warbler	*Abroscopus superciliaris*	12	2.7 ± 1.2	0.09	0.26
GTBA	Grey‐throated Babbler	*Stachyris nigriceps*	10	1.4 ± 0.5	0.09	0.20
BNMO*	Black‐naped Monarch	*Hypothymis azurea*	8	1.5 ± 0.5	0.23	0.00
COTA	Common Tailorbird	*Orthotomus sutorius*	6	2.8 ± 1.7	0.06	0.11
SUTI	Sultan Tit	*Melanochlora sultanea*	6	1.3 ± 0.5	0.09	0.09
YBRWA	Yellow‐browed Warbler	*Phylloscopus inornatus*	6	1.9 ± 0.9	0.11	0.03
HBFL^a^	Hainan Blue Flycatcher	*Cyornis hainanus*	5	1.2 ± 0.5	0.14	0.00
PTBU	Puff‐throated Bulbul	*Alophoixus pallidus*	5	3.0 ± 1.4	0.06	0.09
WBPI	White‐browed Piculet	*Sasia ochracea*	4	1.3 ± 0.5	0.06	0.06

^a^ Species are summer visitors to the region.

**Figure 1:**
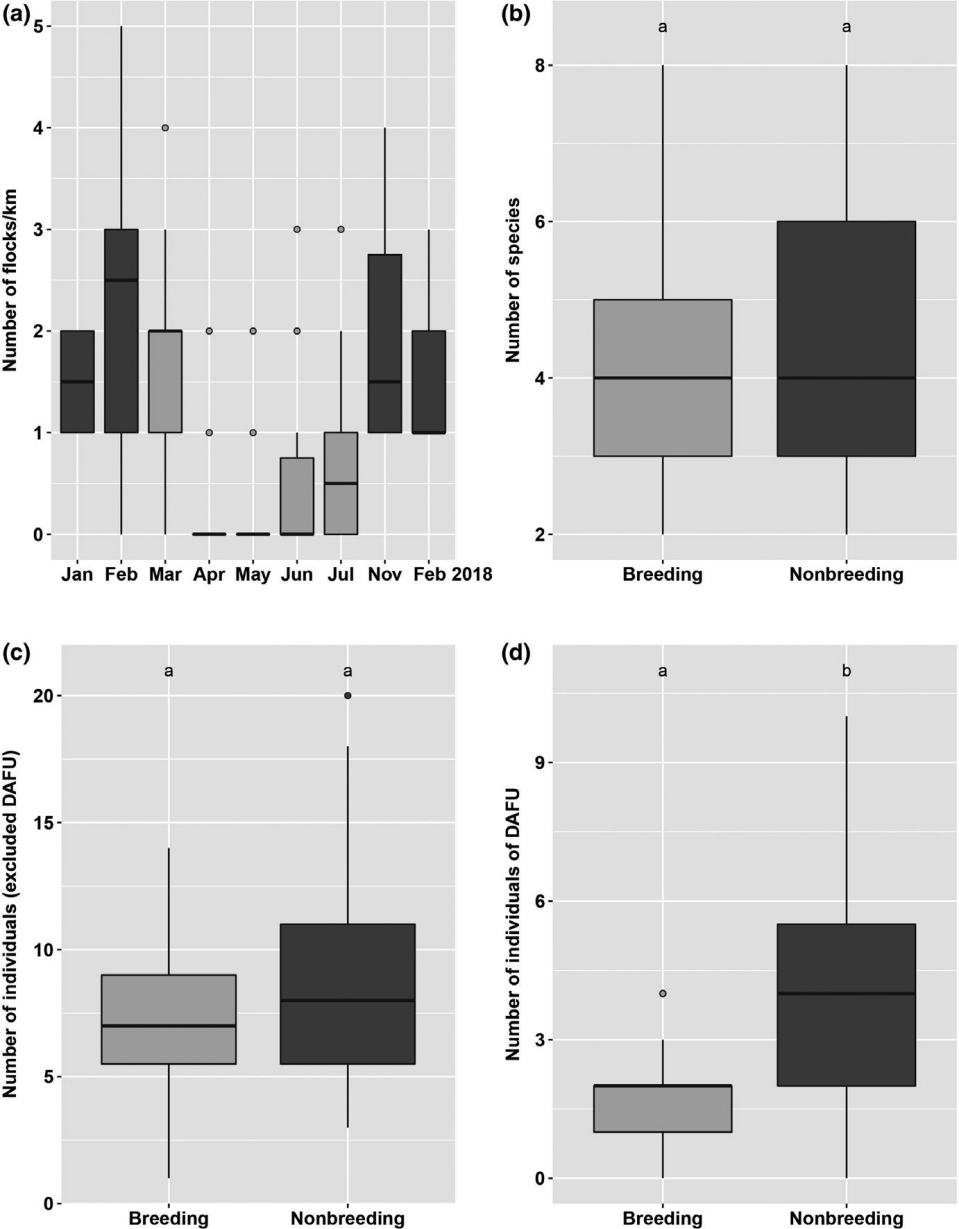
The characteristics of mixed‐species flocks (MSFs) in the different seasons. (a) The number of MSFs encountered per transect walk, over the different months of the survey (January to July 2017, November 2017 and February 2018). (b) The number of species in MSFs in breeding (March to July, light shading) and nonbreeding season (dark shading). (c) The number of individuals not including those of David's Fulvetta (DAFU). (d) The number of individuals of DAFU. The box plots show median values (middle of boxes), lower and upper quartiles (top and bottom of boxes), minimum and maximum values, excluding outliers (the whiskers); points above or below the whiskers are outliers defined as being more than 1.5X the interquartile range (upper minus lower quartiles) away from the lower or upper quartile. Significance of comparisons shown by lettering, with species that were not significantly different (*p* < .05) having the same letter

Season affected MSF encounter rate dramatically (for the comparison between breeding and nonbreeding seasons, Table 1, Figure 1a). MSF encounter rates decreased from a median of two MSFs per visit in the nonbreeding season to a median of zero at the height of the breeding season (April and May); intermediate MSF encounter rates were found in early (March) and late (July) breeding season. However, season did not affect the number of flocking species (Figure 1b). The number of individuals of species other than David's Fulvetta was only mildly affected by season (Figure 1c), with a tendency to decrease in the breeding season. But the number of individuals of David's Fulvetta was strongly affected by season and substantially lower in the breeding season (Figure 1d).

### 3.2 Hawk‐flock experiment

We performed 55 experiments in total, of which only 16 were in the breeding season, because MSFs were rare at that time and also less cohesive, making the experiment difficult to perform. Although 11 species were present in MSF in at least 10 trials, only David's Fulvetta and the Pin‐striped Babbler vocally responded more than 10 times, and only David's Fulvetta vocally responded at least five times in both seasons (Table 3). The number of species that vocally responded per trial (1.30 ± 1.10 species) did not change seasonally (Table 1).

**Table 3:** The frequency with which species responded to the three experimental predator simulations and their seasonal change in response. The list includes only species that were present in at least 10 trials; statistical analyses about seasonality were only run if a species was present (for response probability) or responded (for the characteristics of vocal responses) in at least five trials in each season^a^. Number in parentheses indicates the following: (a) for the hawk‐flock experiment, the percentage of trials in which the species was present before the trial and then vocally responded; (b) for the mob‐flock experiment, first, the percentage of trials in which the species was present and approached, and second, the percentage of those responses in which they also vocally responded; (c) for the mob experiment, the percentage of approaches in which they were also vocal. For seasonal change in the characteristics of the responses (response probability [for experiments in flocks only], latency and duration), we show *p*‐values for single‐factor models. The “Response Percentage” for the mob experiment represents the results (*p*‐value) of a Fisher’s exact test on the difference between the seasons in the proportion of trials in which the species responded. Shaded values show that the characteristic was greater in the breeding season than it was in the nonbreeding season; the reverse is true for not‐shaded values. The species are ordered top‐to‐bottom by their frequency in MSFs (Table 2; see that table also for species abbreviations)

Species	Total responses (%)	Hawk‐flock experiment			Total responses	Mob‐flock experiment			Total responses (%)	Mob experiment		
		Seasonal change				Seasonal change				Seasonal change		
		Response, prob (*p*)	Latency (*p*)	Duration (*p*)		Response, prob (*p*)	Latency (*p*)	Duration (*p*)		Response % (*p*)	Latency (*p*)	Duration (*p*)
DAFU	33 (80%)	.09	.37	.84	41 (93%, 93%)	.87	.84	.20	33 (85%)	1.00	.08	.04
PSTB	12 (32%)	.94	–	–	12 (32%, 19%)	.20	–	–	30 (46%)	1.00	.49	.001
RCBA	2 (11%)	.25	–	–	8 (32%, 4%)	.25	–	–		–	–	–
WBER	9 (39%)	.17	–	–	19 (52%, 52%)	.48	.79	.97	21 (32%)	.29	.09	.77
LLWA	1 (5%)	.42	–	–	1 (7%, 7%)	.61	–	–		–	–	–
YBEWA	4 (29%)	–	–	–	11 (64%, 76%)	.37	.19	.80	17 (26%)	1.00	.54	.52
BNMO	–	–	–	–		–	–	–	16 (25%)	<.001	–	–
SUTI		–	–	–		–		–	11 (91%)	<.001	–	–
FTSU^b^	–	–	–	–	18 (NA, 41%)	.015^c^	.91	.66	39 (61%)	.011	.52	.20

^a^ SBSB, CCWA, BIWA, and BLWA (*Phylloscopus reguloides*) were present in more than 10 trials of the hawk–flock experiment and WTFA (*Rhipidura albicollis*) was present in more than 10 trials in both the hawk–flock and mob‐flock experiment, but none of these species responded (by approach or vocalization) more than once, so they were excluded from the table.

^b^ FTSU is a non‐flocking species that was not present in MSFs before the trials.

^c^ We calculated the approach probability with Fisher’s exact test, because FTSU is a nonflocking species.

When species were analyzed together, seasonality did not affect vocal response probability, or latency and duration of vocal response (Table 1). Species identity, however, showed a significant effect on all three response variables. David's Fulvetta vocally responded the most, with a higher approach probability than all other 10 species (*z*‐values > 3.2, *p* < .051). David's Fulvetta also responded the quickest to the moving stick (1.58 ± 1.79 s after throw) and with longest duration (16.67 ± 13.59 s), significantly faster and longer than Pin‐striped Tit‐babbler (both *t*‐values >4.1, *p* < .001).

When species were analyzed separately (Table 3), David's Fulvetta had a tendency to vocally response less in the breeding season, but did not change its latency and duration of vocal response. Other species did not change their probability to vocally respond.

### 3.3 Mob‐flock experiment

Seven trials of control experiments were conducted, but these received no response (in approaches or vocalizations) at all. For the experimental owl treatment, 43 trials were conducted, of which 19 were in the breeding season. Seven species in total were present in at least 10 experimental trials, but only four species responded by approaching the speaker in at least 10 trials (Table 3), including Fork‐tailed Sunbird, a nonflocking species, which was never present before trials, but did come to the owl presentation. Seasonality tended to affect the number of species that approached per trial (Table 1), with more species tending to approach during the breeding season (4.89 ± 1.82) than during the nonbreeding season (3.96 ± 1.73).

**Figure 2:**
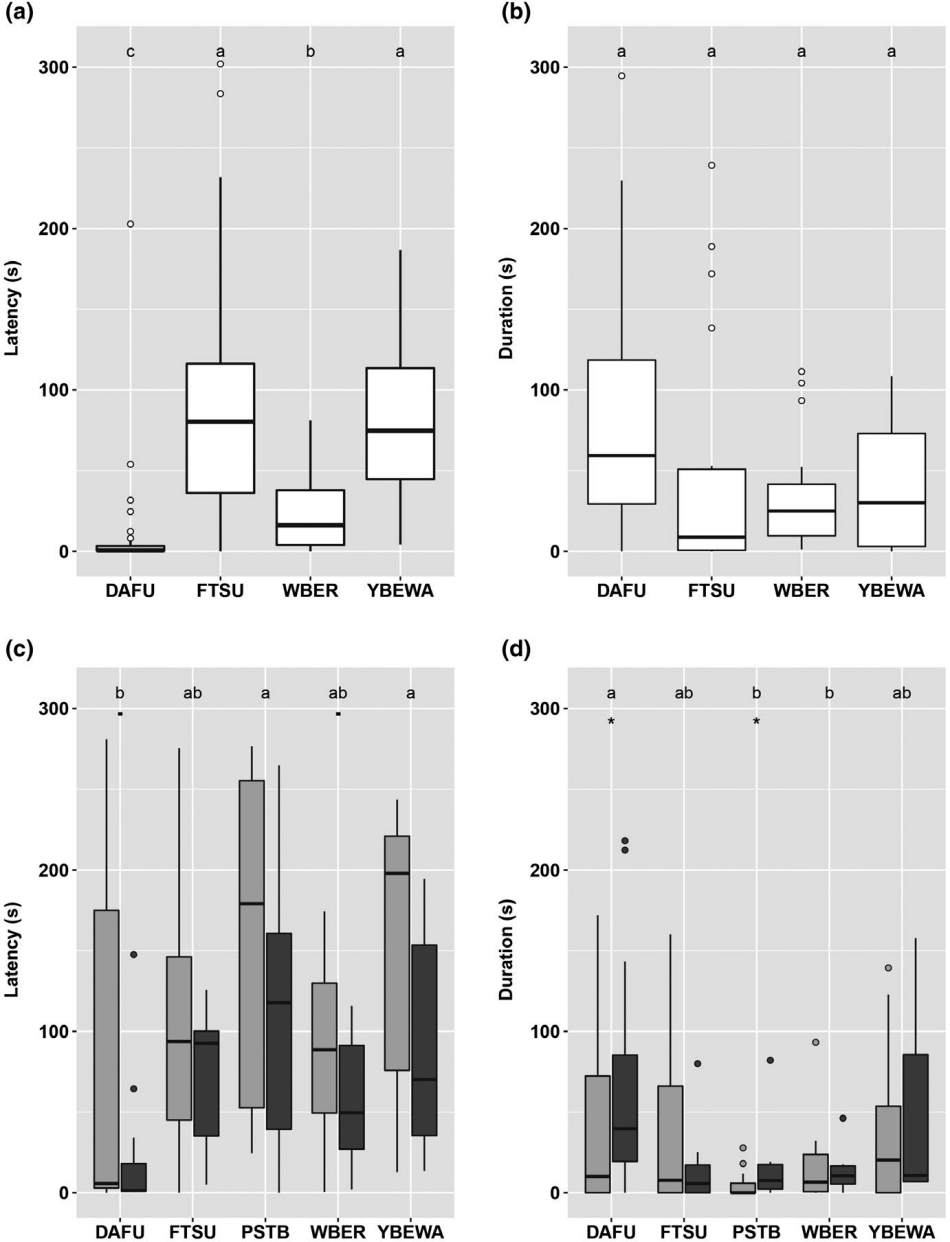
The characteristics of the vocal responses to the stationary predator. Responses inside mixed‐species flocks (MSF) (a, b) did not vary by season, whereas responses outside of MSF (c, d) did change seasonally (breeding season is darkly shaded). Characteristics measured were latency (a, c; time since the stimulus), and duration (b, d; time from start to end of alarm). Significance of comparisons shown by lettering, with species that were not significantly different having the same letter. Symbols in panels c and d denote species that changes in the characteristics of their responses seasonally: • = *p* > .05 and ≤.10, *= *p* > .01 and ≤.05. Species abbreviations as in Table 1; box plots as in Figure 1

When species were analyzed together, seasonality tended to affect the approach probability of MSF participants, with species again tending to approach more in the breeding season. In contrast, seasonality did not affect the latency of vocal response, nor its duration. Similar to the hawk‐flock experiment, however, species identity affected all three response variables (Figure 2, Table 1). Comparing the species, David's Fulvetta had the highest probability to approach the speaker, significantly greater than all other species (six *Z*‐values <3.0, *p* < .029). The fulvetta also had the shortest vocal latency, significantly different than the second‐quickest responder, White‐bellied Erpornis (Figure 2a). David's Fulvetta also tended to have longer vocal duration than White‐bellied Erpornis and Fork‐tailed Sunbird (Figure 2b).

When species were analyzed separately, no species showed a seasonal change in the probability of approaching or the characteristics of its vocal responses (Table 3). However, although the probability of approach for Fork‐tailed Sunbird could not be rated similarly to other species, the commonness with which they responded was higher in the breeding season (12 of 19 MSF) than in the nonbreeding season (6 of 24 MSF, two‐tailed Fisher's exact test, *p* = .016). Further there was one other seasonal difference in vocal responses: David's Fulvetta had considerably fewer (*p* = .005) vocally responding individuals in the breeding season than in the nonbreeding season.

### 3.4 Mob experiment

In 20 trials of the control experiment, again there were no responses (approaches or vocalizations). For the experimental owl treatments, we completed 170 trials in total, of which 28 were in the nonbreeding season (the high number in the breeding season was due to the lesser ability to do MSF‐based experiments in that season). To balance the sample sizes between the seasons, we randomly selected 36 trials conducted in the breeding season, ensuring that each one was done from a different transect visit. In the resultant dataset, seven species responded more than 10 trials (Table 3). There was an average of 3.33 ± 1.66 species responding per trial, significantly less than the mob‐flock experiment (χ^2^_1_ = 13.2, *p* < .001). Seasonality did not affect the number of species that responded.

Mobbing rates (the percentage of all trials in which the species responded) generally increased in the breeding season: six of seven species mobbed in a higher percentage of trials in the breeding season (see Table 3). However, only two species, the summer visitor Black‐naped Monarch (*Hypothymis azurea*) and the Fork‐tailed Sunbird, showed significantly higher mobbing rates in the breeding season. In contrast, one species, the Sultan Tit, reduced its mobbing rates in the breeding season.

When species were analyzed together in the characteristics of their vocal responses, seasonality affected latency but not duration, with all species increasing latency in the breeding season. Species identity affected both vocal latency and duration. David's Fulvetta had a lower latency than two species, and a tendency to be lower than a third species (Figure 2c). Similarly, David's Fulvetta had longer duration than two species, and a tendency to be longer than a third species (Figure 2d).

When species were analyzed separately in the characteristics of their vocal responses, the seasonal change was also evident (Table 3). Two species tended to have longer latencies in the breeding season (Figure 2c), and two species decreased in their duration in the breeding season (Figure 2d). There was no significant difference between seasons in the number of individuals of David's Fulvetta that we estimated vocally responded.

## 4 DISCUSSION

### 4.1 Seasonality of the Nonggang MSF system

In some truly tropical systems, MSFs may continue right through the breeding season: for example, Munn (1984) described nesting birds traveling back and forth to MSFs. However, the MSF system in our study site is more like a temperate one than a tropical one, with a strong decline in encounter rate (though not species richness) in the breeding period. Some of this change may be resource driven. MSFs can decline when supplemental food is experimentally provisioned (Berner & Grubb, 1985; Grubb, 1987; Kubota & Nakamura, 2000; Székely, Szép, & Juhász, 1989), and birds tend to use MSF more often in poor conditions (Gentry et al., 2019; Mangini & Areta, 2018). Develey and Peres (2000) found that in a seasonal tropical forest, the size of MSFs negatively correlated with food resource abundance: that is, MSF size was smaller in the breeding season when arthropod abundance was higher. In our study site, the diversity of fauna and flora are seasonal (You, Li, & Li, 1982; Zhou, Wei, Li, Huang, & Luo, 2006), with insects increasing in the breeding season, and the abundance and richness of soil fauna also higher in the breeding season (Aiwu Jiang, unpublished data). Hence, the seasonal decline of encounter rate of MSFs in the breeding season that we found here could be related to greater resource availability and less variability. Another factor could be that adult survival may be higher in the breeding season than the nonbreeding season, influencing flocking propensity (Morse, 1970); however, we do not think this factor is so important in tropical climates without extreme cold events.

Birds’ social organization is also affected by their movement patterns around nests and young. Flocks were particularly rare in the months of April and May when birds were making nests, incubating, and nestling were not yet fledged. What is interesting is that in MSFs encountered during the breeding season the number of individuals of following species was the same as usual, but there were fewer individuals of David's Fulvettas (Figure 1c,d). To us, this suggests that following species were ready to flock if a flock was present, but that the number of available David's Fulvettas was the factor that limited flock presence. Although David's Fulvetta is highly gregarious, as mentioned in the introduction, they do not seem to be cooperative breeders, and indeed this fieldwork we made observations of four nests and only saw two birds attending each one. Therefore, the transformation in social organization for David's Fulvetta is quite dramatic, going from many individuals in flocks to a pair of breeding birds near the nest. Future research needs to identify what individuals are involved in those rare flocks that do form during the breeding season, and whether these are nonbreeding individuals (i.e., “floaters”).

Our results are consistent with previous studies that have shown disruptions in flocking when a nuclear species is experimentally removed. Specifically, after removal other species had lower propensity to MSF (Dolby & Grubb, 1998), or stayed in less risky microhabitats (Martínez et al., 2018). These changes are probably due to a lack of information, specifically about predators, that is normally provided by the nuclear species for these systems (Martínez, Gomez, Ponciano, & Robinson, 2016, respectively; Sullivan, 1984), and we argue below that David's Fulvetta is especially important in the production of information for our study system.

### 4.2 Dominance of David's Fulvetta in information provisioning

Species identity consistently had a highly significant effect on the characteristics of vocal responses. We found David's Fulvetta was critically important for information provision in this MSF system, as it was the species that responded most commonly, by vocalizing in the hawk‐flock experiment and approaching in the mob‐flock experiment, and vocally responded most quickly and for the longest amount of time, in both experimental types. It even retained this position outside of MSFs, approaching during the mob experiment (although in such circumstances the characteristics of its vocal responses were not significantly more than all other species).

In contrast, there were several common species in MSF that hardly responded at all (e.g., Rusty‐capped Babbler, Limestone Leaf Warbler), or responded only moderately (Pin‐striped Tit‐babbler and White‐bellied Erpornis). Further, there were a number of species that responded to the stationary predator quite commonly and aggressively that were not common flocking species (e.g., Sultan Tit, Black‐naped Monarch, and Yellow‐bellied Warbler). One species, the Fork‐tailed Sunbird, is almost never found in MSF, but approached the model during the presentation of the stationary predator to mob. In general, the species that were most active mobbers tended to be among the smallest sized birds in the forest, and hence probably prey species for the small owl (Courter & Ritchison, 2012; Dutour et al., 2017).

In this study, David's Fulvetta was basically the only responder to the hawk‐flock experiment, and thus the communication network is highly asymmetric. This system is thus similar to the temperate system described by Sullivan (1984), and differs from the results of Goodale and Kotagama (2005), in which multiple species alarm called to a moving object stimulus in a Sri Lankan tropical MSF system. Goodale and Kotagama (2005) suggested that the first responder in their system, the Orange‐billed Babbler (*Turdoides rufescens*), was an unreliable alarm caller, and basically a motion detector, making many false alarms to large or fast‐moving nonpredators and that other species compensated for this by producing their own alarm calls for their conspecific audience when there was a real predator. In this study, we found David's Fulvetta to be remarkably sensitive to potential predators, something that has been remarked on before (Chen & Hsieh, 2002) in a closely related species, Taiwan Fulvetta (*Alcippe morrisonia*) that was once thought to be the same species (Zou, Chuan Lim, Marks, Moyle, & Sheldon, 2007). Fulvettas made the same call type when they first caught sight of the human observers, as well as during an actual attack by an Accipiter hawk, as they did to the moving object in the flock‐hawk experiment, although the vocal response to the actual attack was much longer (the hawk made repeated flights through the area). Perhaps then, the lack of vocal alarm calls of other species, means that the information provided by David's Fulvettas is sufficient for them, although this is a hypothesis that requires further testing.

A possible explanation for why nuclear species like David's Fulvetta produce many calls associated with danger is because they tend to be intraspecifically gregarious (Goodale & Beauchamp, 2010). Higher numbers of kin in MSFs would increase the net benefit of alarm calling (Maynard Smith, 1965). Interestingly, at our study site, David's Fulvettas are not so highly gregarious—at least in comparison to Taiwan's Fulvetta, described by Chen and Hsieh (2002) as averaging 32.5 individuals per flock. Indeed, other species in our MSF system (e.g., Pin‐striped Tit‐babbler) where almost as gregarious but did not produce many vocalizations associated with danger. Why exactly David's Fulvetta is so sensitive to disturbance remains an important topic of research, but perhaps its vigilance about predators also underlies its high activity in mobbing outside of MSFs. Our finding that a nuclear species in MSFs was also important in mobbing assemblages is consistent with earlier studies, particularly in North America (Nolen & Lucas, 2009; Turcotte & Desrochers, 2002). Indeed, MSF leaders might be considered “community informants” for the entire avian community (Hetrick & Sieving, 2012).

### 4.3 Seasonal availability of information

When we were developing this project, we hypothesized that if information from fulvettas were to decrease, other species might compensate by increasing their own information in order to keep their conspecifics informed. This idea of species producing their own calls when the information provided by the nuclear species was inadequate was suggested by Goodale and Kotagama (2005) in a slightly different context, as explained in Section 4.2. Our evidence is consistent with this hypothesis, both in decreases in the information produced by fulvettas, and in increases in information produced by other species, but as we argue below, our methods were not sufficient to falsify competing hypotheses that may produce a similar pattern.

As to the seasonal change in information produced by David's Fulvettas, first and foremost, we show that the MSF system centered around this species declines in its frequency during the breeding season, and hence the fulvettas’ role in information production in the forest overall is diminished. We also found that in the breeding season David's Fulvetta tended (*p* = .09) to respond less in the hawk‐flock experiments. We should remember that this result is at the species‐level: the fact that this species has many individuals per flock might conceal declines, because even if one individual responded, the whole species is rated at responding (and, as mentioned in the methods, in the hawk‐flock experiment the response was so nearly instantaneous that we did not try to estimate how many individuals were involved). In the mob‐flock experiment, we showed a substantial drop in the breeding season in the number of individual fulvettas that vocally responded, and this is probably linked to there simply being fewer fulvettas in flocks at that time (Figure 1d). Having fewer individuals means there are fewer eyes to detect an incoming threat, which can lead to a less rapid or informative response (e.g., figure 3 in Goodale & Kotagama, 2005; although we acknowledge we were not able to see such an effect in this study, perhaps because the small dataset was not collected with this aim in mind).

As to seasonal change in the information production of species other than David's Fulvetta, our results showed a general, although not universal, increase in information production during the breeding season. Species increased in the breeding season their approaches to the mob‐flock experiment: season was a significant influence in that two‐factor analysis. In the mob experiment, 6/7 species had higher mean mobbing activity in the breeding season, though season was not there significant (when analyzing a larger number of trials in the breeding season, however, we find there is a significant increase, Jiang et al., manuscript in preparation).

An alternative hypothesis as to why species other than fulvettas increase their information production in the breeding season is that they have greater investment in that season in their young, including increased nest defense and the need to inform or teach young about potential threats (Curio, 1978; Griesser & Suzuki, 2017; Ibanez‐Alamo et al., 2015). As mentioned in the introduction, several articles have found mobbing in single species to peak in the breeding season; Zimmermann and Curio (1988), for instance, found that great tits (*Parus majors*) approached an owl model and called significant earlier when they had young in the nest, compared to the nonbreeding season or when they were building nests. Mobbing behavior can vary within a given season according to female fertility (Bērziņš et al., 2010) and nest stage. For example, Barn Swallows (*Hirundo rustica*) displayed a low intensity of mobbing during prenesting stages, increased that to a high level when their first brood was in the nest, but then were less active when they had their second broods and thereafter, eventually decreasing to the lowest level of mobbing activity postnesting (Smith & Graves, 1978).

In this study, we made only the coarse comparison between the nonbreeding season and breeding season; future work we hope will focus on changes between different stages of nesting. Also, we hope that future research can attempt to distinguish the compensation hypothesis from the offspring‐investment hypothesis to better understand seasonal changes in information production. Two aspects of our results suggest that offspring investment might be more likely. First, 5/5 species showed higher mean latencies in the breeding season, and 4/5 showed shorter mean durations (with two species showing significant changes in duration; the two variables may be related as a slower responding individual cannot call for as long as a more rapidly responding one). These differences could be linked to changes in movement patterns, or changes in propensity to mob, associated with nesting. Perhaps birds need to travel further from nests, or are more risk adverse when they are nesting, and hence make shorter calls, as long‐lasting mobbing calls have been shown to attract predators (Krams, Krama, Igaune, & Mand, 2007). Another result that makes the offspring investment hypothesis more compelling than the compensation hypothesis for this system is that one of the species that showed the largest seasonal changes, the Fork‐tailed Sunbird, is not a flocking species, and hence would not be expected to be particularly reliant on information from David's Fulvettas.

## 5 CONCLUSIONS

MSFs in this seasonal tropical forest became rarer during the breeding season, synchronous with the nuclear species, David's Fulvetta, breaking into nesting pairs. The decline of the flock system in the breeding season, and the responses to our experimental simulations in those flocks that remained, show that vocal information production by fulvettas is seasonally dynamic. Nevertheless, our data also demonstrate that these fulvettas remain a disproportionately important source of information for the bird community throughout the year. Although fulvettas tended to call less in the hawk‐flock experiment in the breeding season, they were still the only species to call more than five times in both seasons in that experiment. Also, although fewer fulvetta individuals called to the stationary predator model presented in MSFs in the breeding season, fulvettas remained the most frequent approaching species, and the quickest and long‐lasting vocal respondents during that season. In both seasons, fulvetta were among the most common and active mobbers outside of MSFs. Nuclear species of MSF systems have been suggested to be good targets of conservation because other species are reliant on them (Zou et al., 2018). A better understanding of information flow in MSFs, and its behavioral underpinnings, could further make the case that David's Fulvettas are a key species to conserve in management strategies for the birds of south China.

## 6 PERMISSIONS AND PROTECTION OF ANIMALS IN RESEARCH

The experiments were conducted on free‐living birds in the natural habitat, with permission granted by the Nonggang National Nature Reserve. We also designed the methodology to adhere to the ASAB/ABS Guidelines for the Use of Animals in Research, visiting transects only twice per month and conducting at most three playbacks on each visit. The response to the experiments generally ended shortly after playback (a maximum of 5 min).

## DISCLOSURE STATEMENT

The authors declare no conflicts of interest.

## AUTHOR CONTRIBUTION

**Demeng Jiang**: Conceptualization (equal); Formal analysis (lead); Investigation (lead); Methodology (lead); Software (lead); Visualization (lead); Writing‐original draft (equal); Writing‐review & editing (equal). **Kathryn E. Sieving**: Formal analysis (supporting); Validation (supporting); Writing‐original draft (equal); Writing‐review & editing (equal). **Estelle Meaux**: Formal analysis (supporting); Investigation (supporting); Software (supporting); Writing‐original draft (equal); Writing‐review & editing (supporting). **Eben Goodale**: Formal analysis (supporting); Methodology (equal); Supervision (lead); Validation (lead); Writing‐original draft (equal); Writing‐review & editing (equal).

## AUTHORS’ CONTRIBUTIONS

DJ and EG: Idea development; DJ: Fieldwork. All the authors: Analysis and write‐up.

## Data Availability

The data are accessible on Dryad at https://doi.org/10.5061/dryad.rfj6q5777.

## References

[ece36280-bib-0001] Bates, D. , Maechler, M. , Bolker, B. , & Walker, S. (2015). Fitting linear mixed‐effects models using lme4. Journal of Statistical Software, 67, 1–48.

[ece36280-bib-0002] Berner, T. O. , & Grubb, T. C. Jr (1985). An experimental analysis of mixed‐species flocking in birds of deciduous woodland. Ecology, 66, 1229–1236. 10.2307/1939176

[ece36280-bib-0003] Bērziņš, A. , Krama, T. , Krams, I. , Freeberg, T. M. , Kivleniece, I. , Kullberg, C. , & Rantala, M. J. (2010). Mobbing as a trade‐off between safety and reproduction in a songbird. Behavioral Ecology, 21, 1054–1060. 10.1093/beheco/arq104

[ece36280-bib-0004] Charnov, E. L. , & Krebs, J. R. (1975). The evolution of alarm calls: Altruism or manipulation? American Naturalist, 109, 107–112. 10.1086/282979

[ece36280-bib-0005] Chen, C.‐C. , & Hsieh, H. (2002). Composition and foraging behaviour of mixed‐species flocks led by the Grey‐cheeked Fulvetta in Fushan Experimental Forest, Taiwan. Ibis, 144, 317–330. 10.1046/j.1474-919X.2002.00020.x

[ece36280-bib-0006] Courter, J. R. , & Ritchison, G. (2012). Asymmetries in mobbing behavior among nuclear flockmates. Wilson Journal of Ornithology, 124, 626–629. 10.1676/11-168.1

[ece36280-bib-0007] Curio, E. (1978). The adaptive significance of avian mobbing. I. Teleonomic hypotheses and predictions. Zeitschrift Fur Tierpsychologie, 48, 175–183.

[ece36280-bib-0008] Dagan, U. , & Izhaki, I. (2019). The effect of pine forest structure on bird‐mobbing behavior: From individual response to community composition. Forests, 10, 762 10.3390/f10090762

[ece36280-bib-0009] Dall, S. R. X. , Giraldeau, L.‐A. , Olsson, O. , McNamara, J. M. , & Stephens, D. W. (2005). Information and its use by animals in evolutionary ecology. Trends in Ecology and Evolution, 20, 187–193. 10.1016/j.tree.2005.01.010 16701367

[ece36280-bib-0010] Danchin, E. , Giraldeau, L.‐A. , Valone, T. J. , & Wagner, R. H. (2004). Public information: From nosy neighbors to cultural evolution. Science, 305, 487–491. 10.1126/science.1098254 15273386

[ece36280-bib-0011] Develey, P. F. , & Peres, C. A. (2000). Resource seasonality and the structure of mixed species bird flocks in a coastal Atlantic forest of southeastern Brazil. Journal of Tropical Ecology, 16, 33–53. 10.1017/S0266467400001255

[ece36280-bib-0012] Dolby, A. S. , & Grubb, T. C. Jr (1998). Benefits to satellite members in mixed‐species foraging groups: An experimental analysis. Animal Behaviour, 56, 501–509. 10.1006/anbe.1998.0808 9787042

[ece36280-bib-0013] Dutour, M. , Cordonnier, M. , Léna, J.‐P. , & Lengagne, T. (2019). Seasonal variation in mobbing behaviour of passerine birds. Journal of Ornithology, 160, 509–514. 10.1007/s10336-019-01630-5

[ece36280-bib-0014] Dutour, M. , Lena, J.‐P. , & Lengagne, T. (2017). Mobbing behaviour in a passerine community increases with prevalence in predator diet. Ibis, 159, 324–330. 10.1111/ibi.12461

[ece36280-bib-0015] Farine, D. R. , Aplin, L. M. , Sheldon, B. C. , & Hoppitt, W. (2015). Interspecific social networks promote information transmission in wild songbirds. Proceedings of the Royal Society B: Biological Sciences, 282(1803), 20142804, 10.1098/rspb.2014.2804 PMC4345451 25673683

[ece36280-bib-0016] Flower, T. P. , Gribble, M. , & Ridley, A. R. (2014). Deception by flexible alarm mimicry in an African bird. Science, 344, 513–516. 10.1126/science.1249723 24786078

[ece36280-bib-0017] Gentry, K. E. , Roche, D. P. , Mugel, S. G. , Lancaster, N. D. , Sieving, K. E. , Freeberg, T. M. , & Lucas, J. R. (2019). Flocking propensity by satellites, but not core members of mixed‐species flocks, increases when individuals experience energetic deficits in a poor‐quality foraging habitat. PLoS ONE, 14, e0209680 10.1371/journal.pone.0209680 30625186 PMC6326460

[ece36280-bib-0018] Gil, M. A. , Hein, A. M. , Spiegel, O. , Baskett, M. L. , & Sih, A. (2018). Social information links individual behavior to population and community dynamics. Trends in Ecology and Evolution, 33, 535–548. 10.1016/j.tree.2018.04.010 29748042

[ece36280-bib-0019] Goodale, E. , & Beauchamp, G. (2010). The relationship between leadership and gregariousness in mixed‐species bird flocks. Journal of Avian Biology, 41, 99–103. 10.1111/j.1600-048X.2009.04828.x

[ece36280-bib-0020] Goodale, E. , Beauchamp, G. , Magrath, R. , Nieh, J. C. , & Ruxton, G. D. (2010). Interspecific information transfer influences animal community structure. Trends in Ecology and Evolution, 25, 354–361. 10.1016/j.tree.2010.01.002 20153073

[ece36280-bib-0021] Goodale, E. , Beauchamp, G. , & Ruxton, G. D. (2017). Mixed‐species animal groups: Behavior, community structure and conservation. London: Academic Press.

[ece36280-bib-0022] Goodale, E. , & Kotagama, S. W. (2005). Alarm calling in Sri Lankan mixed‐species bird flocks. The Auk, 122, 108–120. 10.1093/auk/122.1.108

[ece36280-bib-0023] Goodale, E. , & Kotagama, S. W. (2006). Vocal mimicry by a passerine bird attracts other species involved in mixed‐species flocks. Animal Behaviour, 72, 471–477. 10.1016/j.anbehav.2006.02.004

[ece36280-bib-0024] Goodale, E. , Nizam, B. Z. , Robin, V. V. , Sridhar, H. , Trivedi, P. , Kotagama, S. W. , … Vijayan, L. (2009). Regional variation in the composition and structure of mixed‐species bird flocks in the Western Ghats and Sri Lanka. Current Science, 97, 648–663.

[ece36280-bib-0025] Goodale, E. , Sridhar, H. , Sieving, K. E. , Bangal, P. , Colorado Z., G. J. , Farine, D. R. , … Shanker, K. (2020). Mixed company: A framework for understanding the composition and organization of mixed‐species animal groups. Biological Reviews. 10.1111/brv.12591 PMC7383667 32097520

[ece36280-bib-0026] Griesser, M. , & Suzuki, T. N. (2017). Naive juveniles are more likely to become breeders after witnessing predator mobbing. American Naturalist, 189, 58–66. 10.1086/689477 28035889

[ece36280-bib-0027] Grubb, T. C. Jr (1987). Changes in the flocking behaviour of wintering English titmice with time, weather and supplementary food. Animal Behaviour, 35, 794–806. 10.1016/S0003-3472(87)80116-1

[ece36280-bib-0028] Hetrick, S. A. , & Sieving, K. E. (2012). Antipredator calls of tufted titmice and interspecific transfer of encoded threat information. Behavioral Ecology, 23, 83–92. 10.1093/beheco/arr160

[ece36280-bib-0029] Hillemann, F. , Cole, E. F. , Keen, S. C. , Sheldon, B. C. , & Farine, D. R. (2019). Diurnal variation in the production of vocal information about food supports a model of social adjustment in wild songbirds. Proceedings of the Royal Society B‐Biological Sciences, 286, 8 10.1098/rspb.2018.2740 PMC6408885 30963842

[ece36280-bib-0030] Hino, T. (1998). Mutualistic and commensal organization of avian mixed‐species foraging flocks in a forest of western Madagascar. Journal of Avian Biology, 29, 17–24. 10.2307/3677336

[ece36280-bib-0031] Hothorn, T. , Bretz, F. , & Westfall, P. (2008). Simultaneous inference in general parametric models. Biometrical Journal, 50, 346–363. 10.1002/bimj.200810425 18481363

[ece36280-bib-0032] Hua, F. , & Sieving, K. E. (2016). Understory avifauna exhibits altered mobbing behavior in tropical forest degraded by selective logging. Oecologia, 182, 743–754. 10.1007/s00442-016-3695-1 27417548

[ece36280-bib-0033] Huang, P. , Sieving, K. E. , & St Mary, C. M. (2012). Heterospecific information about predation risk influences exploratory behavior. Behavioral Ecology, 23, 463–472. 10.1093/beheco/arr212

[ece36280-bib-0034] Hurd, C. R. (1996). Interspecific attraction to the mobbing calls of black‐capped chickadees (*Parus atricapillus*). Behavioral Ecology and Sociobiology, 38, 287–292. 10.1007/s002650050244

[ece36280-bib-0035] Hutto, R. L. (1994). The composition and social organization of mixed‐species flocks in a tropical deciduous forest in western Mexico. Condor, 96, 105–118. 10.2307/1369068

[ece36280-bib-0036] Ibanez‐Alamo, J. D. , Magrath, R. D. , Oteyza, J. C. , Chalfoun, A. D. , Haff, T. M. , Schmidt, K. A. , … Martin, T. E. (2015). Nest predation research: Recent findings and future perspectives. Journal of Ornithology, 156, S247–S262.

[ece36280-bib-0037] Jayarathna, A. , Kotagama, S. W. , & Goodale, E. (2013). The seasonality of mixed‐species bird flocks in a Sri Lankan rainforest in relation to the breeding of the nuclear species. Orange‐billed Babbler Turdoides Rufescens. Forktail, 138–139.

[ece36280-bib-0038] Jiang, D. , Zhou, F. , Jiang, A. , & Chen, T. (2013). Breeding notes on 18 birds species in limestone area of southwestern Guangxi. Chinese Journal of Zoology, 48, 597–604.

[ece36280-bib-0039] Jones, H. , & Sieving, K. (2019). Foraging ecology drives social information reliance in an avian eavesdropping community. Ecology and Evolution, 9, 11584–11597. 10.1002/ece3.5561 31695870 PMC6822049

[ece36280-bib-0040] Jurisevic, M. A. , & Sanderson, K. J. (1994). Alarm vocalizations in Australian birds – Convergent characteristics and phylogenetic differences. Emu, 94, 67–77.

[ece36280-bib-0041] Klump, G. M. , Kretzschmar, E. , & Curio, E. (1986). The hearing of an avian predator and its avian prey. Behavioral Ecology and Sociobiology, 18, 317–323. 10.1007/BF00299662

[ece36280-bib-0042] Klump, G. M. , & Shalter, M. D. (1984). Acoustic behaviour of birds and mammals in the predator context. I. Factors affecting the structure of alarm calls. II. The functional significance and evolution of alarm calls. Zeitschrift Fur Tierpsychologie, 66, 189–226.

[ece36280-bib-0043] Krams, I. (2001). Communication in crested tits and the risk of predation. Animal Behaviour, 61, 1065–1068. 10.1006/anbe.2001.1702

[ece36280-bib-0044] Krams, I. , Krama, T. , Igaune, K. , & Mand, R. (2007). Long‐lasting mobbing of the pied flycatcher increases the risk of nest predation. Behavioral Ecology, 18, 1082–1084. 10.1093/beheco/arm079

[ece36280-bib-0045] Kubota, H. , & Nakamura, M. (2000). Effects of supplemental food on intra‐and inter‐specific behaviour of the Varied Tit *Parus varius* . Ibis, 142, 312–319. 10.1111/j.1474-919X.2000.tb04871.x

[ece36280-bib-0046] Langham, G. M. , Contreras, T. A. , & Sieving, K. E. (2006). Why pishing works: Titmouse (Paridae) scolds elicit a generalized response in bird communities. Ecoscience, 13, 485–496. 10.2980/1195-6860(2006)13[485:WPWTPS]2.0.CO;2

[ece36280-bib-0047] Mangini, G. G. , & Areta, J. I. (2018). Bird mixed‐species flock formation is driven by low temperatures between and within seasons in a Subtropical Andean‐foothill forest. Biotropica, 50, 816–825. 10.1111/btp.12551

[ece36280-bib-0048] Marler, P. (1955). Characteristics of some animal calls. Nature, 176, 6–8. 10.1038/176006a0

[ece36280-bib-0049] Martínez, A. , Gomez, J. P. , Ponciano, J. M. , & Robinson, S. K. (2016). Functional traits, sociality and perceived predation risk in an Amazonian understory bird community. American Naturalist, 187, 607–619. 10.1086/685894 27104993

[ece36280-bib-0050] Martínez, A. E. , Parra, E. , Muellerklein, O. , & Vredenburg, V. T. (2018). Fear‐based niche shifts in neotropical birds. Ecology, 99(6), 1338–1346. 10.1002/ecy.2217 29787637

[ece36280-bib-0051] Maynard Smith, J. (1965). The evolution of alarm calls. American Naturalist, 99, 59–63. 10.1086/282349

[ece36280-bib-0052] Mönkkönen, M. , & Forsman, J. T. (2002). Heterospecific attraction among forest birds: A review. Ornithological Science, 1, 41–51. 10.2326/osj.1.41

[ece36280-bib-0053] Morse, D. H. (1970). Ecological aspects of some mixed‐species foraging flocks of birds. Ecological Monographs, 40, 119–168. 10.2307/1942443

[ece36280-bib-0054] Moynihan, M. (1962). The organization and probable evolution of some mixed‐species flocks of Neotropical birds. Smithsonian Miscellaneous Collections, 143, 1–140.

[ece36280-bib-0055] Munn, C. A. (1984). The behavioral ecology of mixed‐species bird flocks in Amazonian Peru. PhD thesis, Princeton University, Princeton, NJ.

[ece36280-bib-0056] Munn, C. A. , & Terborgh, J. W. (1979). Multi‐species territoriality in Neotropical foraging flocks. Condor, 81, 338–347. 10.2307/1366956

[ece36280-bib-0057] Nocera, J. J. , Taylor, P. D. , & Ratcliffe, L. M. (2008). Inspection of mob‐calls as sources of predator information: Response of migrant and resident birds in the Neotropics. Behavioral Ecology and Sociobiology, 62, 1769–1777. 10.1007/s00265-008-0605-5

[ece36280-bib-0058] Nolen, M. T. , & Lucas, J. R. (2009). Asymmetries in mobbing behaviour and correlated intensity during predator mobbing by nuthatches, chickadees and titmice. Animal Behaviour, 77, 1137–1146. 10.1016/j.anbehav.2009.01.023

[ece36280-bib-0059] Pavey, C. R. , & Smyth, A. K. (1998). Effects of avian mobbing on roost use and diet of powerful owls, *Ninox strenua* . Animal Behaviour, 55, 313–318. 10.1006/anbe.1997.0633 9480699

[ece36280-bib-0060] Pettifor, R. A. (1990). The effects of avian mobbing on a potential predator, the European kestrel, *Falco tinnunculus* . Animal Behaviour, 39, 821–827. 10.1016/S0003-3472(05)80945-5

[ece36280-bib-0061] Rainey, H. J. , Zuberbühler, K. , & Slater, P. J. B. (2004). Hornbills can distinguish between primate alarm calls. Proceedings of the Royal Society London B, 271, 755–759. 10.1098/rspb.2003.2619 PMC1691652 15209110

[ece36280-bib-0062] Rodewald, A. D. , & Abrams, M. D. (2002). Floristics and avian community structure: Implications for regional changes in Eastern forest composition. Forest Science, 48, 267–272.

[ece36280-bib-0063] Schmidt, K. A. , Dall, S. R. X. , & van Gils, J. A. (2010). The ecology of information: An overview on the ecological significance of making informed decisions. Oikos, 119, 304–316. 10.1111/j.1600-0706.2009.17573.x

[ece36280-bib-0064] Schmidt, K. A. , Lee, E. , Ostfeld, R. S. , & Sieving, K. (2008). Eastern chipmunks increase their perception of predation risk in response to titmouse alarm calls. Behavioral Ecology, 19, 759–763. 10.1093/beheco/arn034

[ece36280-bib-0065] Seppänen, J.‐T. , Forsman, J. T. , Mönkkönen, M. , & Thomson, R. L. (2007). Social information use is a process across time, space and ecology, reaching heterospecifics. Ecology, 88, 1622–1633. 10.1890/06-1757.1 17645008

[ece36280-bib-0066] Shedd, D. H. (1982). Seasonal variation and function of mobbing and related antipredator behaviors of the American Robin (*Turdus migratorius*). The Auk, 99, 342–346.

[ece36280-bib-0067] Shields, W. M. (1984). Barn swallow mobbing: Self‐defence, collateral kin defence, group defence, or parental care? Animal Behaviour, 32, 132–148. 10.1016/S0003-3472(84)80331-0

[ece36280-bib-0068] Smith, J. M. , & Graves, H. B. (1978). Some factors influencing mobbing behavior in Barn Swallows (*Hirundo rustica*). Behavioral Biology, 23, 355–372. 10.1016/S0091-6773(78)91379-2

[ece36280-bib-0069] Sridhar, H. , Beauchamp, G. , & Shanker, K. (2009). Why do birds participate in mixed‐species foraging flocks? A large‐scale synthesis. Animal Behaviour., 78, 337–347. 10.1016/j.anbehav.2009.05.008

[ece36280-bib-0070] Sridhar, H. , & Guttal, V. (2018). Friendship across species borders: Factors that facilitate and constrain heterospecific sociality. Philosophical Transactions of the Royal Society B, Biological Sciences, 373, 20170014 10.1098/rstb.2017.0014 PMC5882984 29581399

[ece36280-bib-0071] Sullivan, K. A. (1984). Information exploitation by downy woodpeckers in mixed‐species flocks. Behaviour, 91, 294–311. 10.1163/156853984X00128

[ece36280-bib-0072] Székely, T. , Szép, T. , & Juhász, T. (1989). Mixed‐species flocking of tits (*Parus* spp.): A field experiment. Oecologia, 78, 490–495. 10.1007/BF00378739 28312178

[ece36280-bib-0073] Templeton, C. N. , & Greene, E. (2007). Nuthatches eavesdrop on variations in heterospecific chickadee mobbing alarm calls. Proceedings of the National Academy of Sciences USA, 104, 5479–5482. 10.1073/pnas.0605183104 PMC1838489 17372225

[ece36280-bib-0074] Tubelis, D. P. , Cowling, A. , & Donnelley, C. (2006). Role of mixed‐species flocks in the use of adjacent savannas by forest birds in the central Cerrado, Brazil. Austral Ecology, 31, 38–45. 10.1111/j.1442-9993.2006.01541.x

[ece36280-bib-0075] Turcotte, Y. , & Desrochers, A. (2002). Playbacks of mobbing calls of Black‐capped Chickadees help estimate the abundance of forest birds in winter. Journal of Field Ornithology, 73, 303–307. 10.1648/0273-8570-73.3.303

[ece36280-bib-0076] You, Q. , Li, T. , & Li, Y. (1982). Survey of Insect fauna and ecnomical insect of Nonggang Nature Reserve. Journal of Guangxi Academy of Sciences, 1, 133–140.

[ece36280-bib-0077] Zhou, F. (1989). Breeding ecology of Grey‐cheecked Fulvetta (*Alcippe morrisonia*). Chinese Journal of Wildlife, 6, 54–56.

[ece36280-bib-0078] Zhou, Q. , Wei, F. , Li, M. , Huang, C. , & Luo, B. (2006). Diet and food choice of *Trachypithecus francoisi* in the Nonggang Nature Reserve, China. International Journal of Primatology, 27, 1441–1460. 10.1007/s10764-006-9082-8

[ece36280-bib-0079] Zimmermann, U. , & Curio, E. (1988). Two conflicting needs affecting predator mobbing by great tits, *Parus major* . Animal Behaviour, 36, 926–932. 10.1016/S0003-3472(88)80175-1

[ece36280-bib-0080] Zou, F. , Chuan Lim, H. , Marks, B. D. , Moyle, R. G. , & Sheldon, F. H. (2007). Molecular phylogenetic analysis of the Grey‐cheeked Fulvetta (*Alcippe morrisonia*) of China and Indochina: A case of remarkable genetic divergence in a “species”. Molecular Phylogenetics and Evolution, 44, 165–174. 10.1016/j.ympev.2006.12.004 17300964

[ece36280-bib-0081] Zou, F. , Jones, H. , Colorado Z., G. J. , Jiang, D. , Lee, T.‐M. , Martínez, A. , … Goodale, E. (2018). The conservation implications of mixed‐species flocking in terrestrial birds, a globally‐distributed species interaction network. Biological Conservation, 224, 267–276. 10.1016/j.biocon.2018.06.004

